# Food Insecurity Moderates the Acute Effect of Subjective Socioeconomic Status on Food Consumption

**DOI:** 10.3389/fpsyg.2019.01886

**Published:** 2019-08-14

**Authors:** Sarah Godsell, Michael Randle, Melissa Bateson, Daniel Nettle

**Affiliations:** Centre for Behaviour and Evolution, Institute of Neuroscience, Newcastle University, Newcastle upon Tyne, United Kingdom

**Keywords:** food insecurity, socioeconomic status, obesity, eating, energy intake

## Abstract

Experimentally inducing low subjective socioeconomic status (SSES) increases food consumption in standardized eating opportunities. Separately, food insecurity (FI) has also been shown to be associated with increased food consumption when a free eating opportunity is provided. Here, we assigned 123 adult volunteers to a low-SSES manipulation or a control condition, followed by an opportunity to consume snack foods. We measured FI prior to the experiment. Thus, our experiment served to replicate the effects of SSES and of FI on consumption, and also to establish whether these effects combine additively or interactively. The low-SSES manipulation increased food consumption, but only among participants who were food secure at baseline. Among food-insecure participants, the effect was reversed. This interaction was not predicted *a priori* and is presented as an exploratory finding. We also found evidence that both SSES and FI affected the hedonic evaluation of the snack foods, though the changes in evaluation did not mediate the changes in consumption. Our findings suggest that both FI and low SSES affect the consumption and evaluation of food. Their combined effects on consumption may be complex.

## Introduction

Low socioeconomic status is a predictor of obesity and overweight in developed countries, especially among women (Sobal and Stunkard, 1989; McLaren, 2007; Newton et al., 2017). Higher energy intake is thought to contribute to this gradient (Jeffery and French, 1996). Subjective socioeconomic status (SSES; the self-assessed appraisal of one’s position within society) explains variation in obesity above and beyond more objective socioeconomic variables (Goodman et al., 2001, 2003). This suggests that, as well as the economic constraints on food choice imposed by low socioeconomic status, there may be fundamental links between the feeling of being disadvantaged and the motivation to eat more (see Bratanova et al., 2016). Recently, researchers have begun to manipulate SSES experimentally, using tasks that cause participants to compare their lot favorably or unfavorably to others. Such manipulations increase intended or actual food consumption in their immediate aftermath (Cardel et al., 2015; Bratanova et al., 2016; Cheon and Hong, 2016; Sim et al., 2018a), and increase secretion of the hormone ghrelin (Sim et al., 2018b). The experimental approach is attractive because it offers the possibility of answering two linked questions definitively: first, whether the feeling of low status is sufficient to affect energy intake, even without any change in objective socioeconomic status; and second, whether the relationship between low SSES and food consumption (and hence, by inference, obesity) is causal.

Food insecurity – the limited or uncertain ability to procure adequate food – also predicts obesity and overweight among women (Townsend et al., 2001; Nettle et al., 2017). In two recent studies, food-insecure participants consumed more calories when given free eating opportunities (Stinson et al., 2018; Nettle et al., 2019). Thus, two factors appear to predict how much a person will eat when given an opportunity: their (experimentally manipulated) SSES; and their (baseline) FI. As these factors have only recently been documented, their effects each require replication. Further, it is currently unknown how SSES and FI might combine in predicting consumption. One possible reason that inducing low SSES increases eating is that it makes people feel as if they were food insecure. In this case, there might be no effect, or a smaller effect, in people who feel food insecure already. On the other hand, baseline FI might sensitize individuals to feelings of low status, meaning that food-insecure individuals show a larger response to acute low SSES than food-secure individuals. Similar sensitization arguments have been made, for example, for low childhood SES (Griskevicius et al., 2013; Hill et al., 2013). In either of these cases, rather than two additive effects, there would be a significant interaction between the SSES manipulation and baseline FI in predicting consumption.

As well as finding that food-insecure women consumed more calories than food-secure women, Nettle et al. (2019) found that food-insecure women rated one of the foods (chocolate) more highly when asked to evaluate it. Thus, an important proximate psychological effect of FI may be to increase the hedonic value of foods, leading to greater consumption, as suggested by Anselme and Güntürkün (2018). Just as FI might lead to changes in the hedonic value of foods, so might SSES. That is, the increased consumption seen following SSES manipulations might be due to changes in the perception of the hedonic value of the foods on offer. This possibility has not been tested in previous studies.

The present study is a conceptual replication of Nettle et al. (2019), in which we measured baseline FI in an opportunity sample, then presented a mock “taste test” of snack foods, and measured energy intake. The innovation of the present study was that we also included an SSES manipulation (following the methods of Cheon and Hong, 2016). Hence, our study also provides a replication of the acute experimental effect of SSES on consumption. Based on prior findings, we made the following confirmatory predictions: there will be an interaction between sex and FI in predicting consumption, with food-insecure women consuming more than food-secure women, but no difference by FI among men; and participants receiving the low-SSES manipulation will consume more than participants in a control condition. We investigated the possibility of interactions between FI and manipulated SSES, but without directional prediction. We also predicted that both FI and SSES would affect the hedonic value of the foods on offer, as reflected in participant ratings, and that these evaluation changes would mediate the changes in consumption. Additionally, we measured positive and negative affect after the manipulation. We present purely exploratory analyses of these variables.

## Materials and Methods

### Ethics Statement

Ethical approval for this study was granted by the Faculty of Medical Sciences Ethics Committee at Newcastle University, approval number 1400/15594/2017. All participants gave written informed consent to participate and were debriefed as to the true nature of the study on completion.

### Data Availability

The raw data from this study, along with R code used for analysis, are freely available via the Zenodo repository at: https://doi.org/10.5281/zenodo.2580973.

### Participants

We recruited an opportunity sample of 125 adult participants (36 males, 89 females; 84 students) via flyers, online advertisement, and registers held at the Newcastle University. Two male participants did not complete key parts of the study, leaving 123 (34 males, 89 females). This gave a power of 0.96 to detect the SSES effect size in Cheon and Hong (2016)’s study 3, and 0.75 to detect the effect size in their study 4. Participants were asked to disclose any food allergies or intolerances that might affect their participation in the “taste test,” and none were excluded for this reason. The study was presented as an investigation of how individual characteristics and eating patterns relate to food preferences.

### Food Insecurity

Participants completed an online questionnaire prior to attending the laboratory, including two measures of FI: the 20-item Adult Food Insecurity (AFI) questionnaire (Nettle et al., 2019) and the widely used 8-item USDA FI scale (Bickel et al., 2000). The AFI focuses on all experiences of FI and irregularity in the past 12 months, regardless of their cause, whereas the USDA, which is the standard FI measure, focuses exclusively on FI due to financial constraints in the last 12 months. For both scales, each item yields a “secure” and “insecure” response, and the score is the number of “insecure” responses. The two FI measures were correlated (*r* = 0.57, *p* < 0.001, with USDA score log transformed), but had very different distributions. For AFI score, only five participants scored zero, and the central tendency was in the middle of the range. For USDA score, 90 participants (73%) scored zero. We retained both measures for analysis, as they may carry somewhat different information: AFI score as a continuous measure of the experience of food irregularity and unpredictability in daily life; and USDA [which we dichotomized as “Secure” (score of 0) or “Insecure” (score > 0)] as the standard marker of serious economically driven FI. We therefore repeat all models first using USDA FI, and then using AFI FI. Retaining two measures of FI increases multiple testing and hence the false positive rate. We hence interpret significant results involving FI with caution (see the section “Discussion”).

The online questionnaire also contained a number of measures of early life experience that are not analyzed here.

### Procedure

Participants were tested singly by one of two experimenters (SG and MR). Assignment to condition was an alternate sign-up basis.

#### Cola Pre-task

On arrival, participants consumed two cups containing 75 g of Coca-Cola^®^ Classic (31.5 kcal) and 75 g of Pepsi (33 kcal), then completed a 10-min filler task in which they watched video advertisements of the two brands and answered questions on their cola preferences. This is a previously used method of equalizing satiety levels prior to the “taste test” (Wang and Dvorak, 2010; Nettle et al., 2019). Participants then rated their current hunger, fullness, and desire to eat on 7-point scales, with 1 representing “not at all” and 7 “extremely.” These three ratings were, after appropriate reversal, all highly inter-correlated (Cronbach’s α = 0.82). Henceforth, they were summed to provide a single variable referred to as current hunger.

#### SSES Manipulation

Participants next completed the experimental manipulation, based on Cheon and Hong (2016), using the “low” and “control” conditions from their study 4. We did not use a “high” SSES condition; in Cheon and Hong (2016) study 4, a “high” was also included, but did not differ significantly from “control.” Each participant was shown an image of a ladder with rungs numbered 1–10, representing UK society. In the low status condition (*n* = 63, 17 male, 46 female), an adjacent body of text asked the participant to compare him/herself to the people at the very top of the latter, think about how he/she was different from those people, and then place him/herself on the ladder relative to them. Following this, the participant was asked to imagine an interaction with a stranger from the top of the ladder. In the control condition (*n* = 60, 17 male, 43 female), participants placed themselves on the ladder without first comparing themselves to the people at the top and no ladder position for the stranger in the imagined interaction was specified. After finishing this, participants completed the PANAS measures of positive and negative affect (Watson et al., 1988).

#### Taste Test

Next, four standard pre-weighed plates of food were simultaneously presented by drawing back curtains. The plates were positioned in a row and contained: milk chocolate buttons (69 g, 516 kcal/100 g); cheese crackers (48 g, 529 kcal/100 g); ready salted potato crisps (50 g, 539 kcal/100 g); and sweet popcorn (35 g, 463 kcal/100 g). The foods were chosen to be highly palatable, energy dense, and offer sweet and savory options. The quantities were chosen to balance available calories from sweet food (518 kcal) and savory food (523 kcal). Water was also presented.

For 5 min, participants were asked to evaluate how much they liked each food on a 7-point scale (1 indicating dislike), adding short qualitative comments indicating what they liked and disliked about it. Once the 5 min had passed, we distributed an additional packet of questionnaires, saying: “Please feel free to eat as much as you’d like as we have to throw the food away between participants.” The experimenter then withdrew for 10 min. Height (stadiometer, 0.5 cm precision) and weight (digital scales, 0.1 kg precision) were recorded on conclusion of the session, in order to calculate body mass index (BMI). Food plates were weighed following the session. Food weights were converted to kilocalories using the nutritional information on the packaging.

### Data Analysis

For consumption, we had four outcome variables (kcals consumed of each of the four foods). We therefore used multivariate analysis of variance (MANOVA) to examine whether FI, experimental SSES condition, sex, and their interactions predicted the set of four consumption outcomes. Sex and its interactions were included as previous studies have found sex differences in the response to FI. For evaluation of the foods, we performed a parallel MANOVA with the four ratings of the foods as the outcomes in place of the kcals consumed. Significant MANOVA findings were followed up with single-outcome general linear models or mediation models where appropriate. All analyses were carried out in R (R Core Development Team, 2018), with an α level of 0.05. The consumption variables had positively skewed distributions and were log-transformed for analysis. Figures are based on untransformed data.

## Results

### Descriptive Statistics

Descriptive statistics for the final study sample are shown in Table 1. The amounts consumed of the different foods were all moderately positively correlated (Cronbach’s α = 0.73), as were the evaluations of the foods (Cronbach’s α = 0.45). The evaluation of the food was moderately correlated with the amount consumed in all cases (chocolate: *r* = 0.37, *p* < 0.001; crackers: *r* = 0.42, *p* < 0.001; crisps: *r* = 0.22, *p* = 0.01; popcorn: *r* = 0.46, *p* < 0.001). Current hunger weakly or negligibly predicted consumption of the foods (chocolate: *r* = 0.12, *p* = 0.18; crackers: *r* = 0.27, *p* = 0.002; crisps: *r* = 0.20, *p* = 0.03; popcorn: *r* = 0.16, *p* = 0.08). Inclusion of current hunger as an additional predictor variable does not affect any of the results presented below.

**TABLE 1:** Descriptive statistics for the main study variables.

**Variable**	**Mean (*SD*) or frequencies**	**Median (range)**
Age	30.66 (16.66)	23 (18–86)
BMI	24.19 (3.78)	23.59 (16.53–36.84)
FI (USDA)	Secure 90	
	Insecure 33	
FI (AFI score)	6.05 (4.11)	6 (0–16)
Current hunger	10.71 (4.01)	10 (3–20)
Chocolate consumption (kcals)	88.34 (84.93)	62.96 (0–362.23)
Cracker consumption (kcals)	51.92 (56.06)	33.86 (0–261.86)
Crisp consumption (kcals)	46.26 (51.00)	30.18 (1.62–239.32)
Popcorn consumption (kcals)	24.78 (33.05)	62.95 (0–162.98)
Evaluation of chocolate	5.21 (1.72)	6 (1–7)
Evaluation of crackers	5.07 (1.47)	5 (1–7)
Evaluation of crisps	5.11 (1.27)	5 (2–7)
Evaluation of popcorn	4.96 (1.56)	5 (1–7)

Food insecurity was randomly distributed across SSES conditions (USDA: χ^2^ = 0.06, *p* = 0.81; AFI: *t* = −0.17, *p* = 0.86). Current hunger did not differ significantly across SSES conditions (*t* = 0.79, *p* = 0.43), and was not related to FI (USDA: *t* = −0.77, *p* = 0.45; AFI: *r* = 0.05, *p* = 0.57). BMI did not differ by SSES condition (*t* = −0.75, *p* = 0.45), and was not related to FI in this sample (USDA: main effect of FI, *t* = 0.57, *p* = 0.57; interaction with sex, *t* = −1.00, *p* = 0.32; AFI: main effect of FI, *t* = 0.51, *p* = 0.21; interaction with sex, *t* = −1.09, *p* = 0.15).

### Calorie Consumption

**FIGURE 1:**
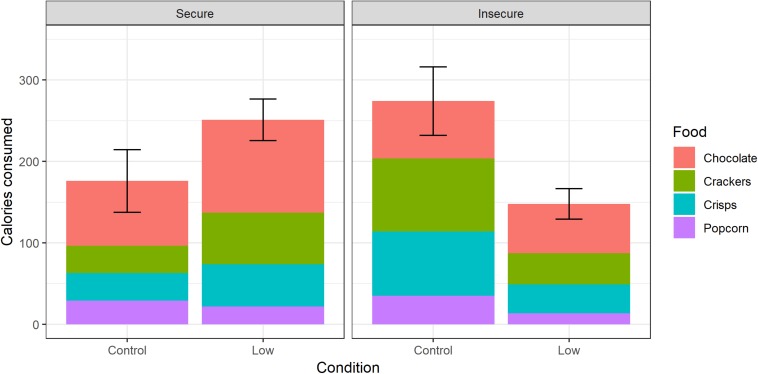
Calorie consumption by food insecurity status (USDA) and experimental SSES condition. Bars are broken into consumption of the four constituent foods. Error bars represent one ± 1 standard error of total consumption.

Table 2 shows MANOVA results. For calorie consumption, using USDA as the FI measure, there was a significant interaction between SSES condition and FI in predicting consumption. To investigate the interaction, we split the data into food-secure and food-insecure participants, and performed separate MANOVAs with condition as the predictor. Among food-secure participants, there was a significant effect of condition (*F*_4__,__85_ = 3.84, *p* = 0.006), with those in the low SSES condition consuming more than those in the control condition (Figure 1). Among food-insecure participants, there was also a significant effect of condition (*F*_4__,__28_ = 2.85, *p* = 0.04), but the difference was in the opposite direction (Figure 1). Splitting into separate MANOVAs by condition instead of FI, in the control condition, there was a significant main effect of FI (*F*_4__,__55_ = 2.80, *p* = 0.03), with food-insecure participants consuming more; whereas in the low SSES condition, the difference between food-secure and food-insecure participants was near-significantly in the opposite direction (*F*_4__,__58_ = 2.40, *p* = 0.06).

**TABLE 2:** MANOVA results.

	**Using USDA FI status**			**Using AFI score**		
		
**Predictor**	***F***	***df***	***p*-value**	***F***	***df***	***p*-value**
**Calorie consumption**						
Condition	3.74	4, 112	0.007^∗^	3.67	4, 112	0.008^∗^
FI	1.15	4, 112	0.33	0.96	4, 112	0.43
Sex	0.32	4, 112	0.87	0.28	4, 112	0.89
Condition * FI	3.46	4, 112	0.01^∗^	0.54	4, 112	0.71
Condition * Sex	2.10	4, 112	0.09	1.88	4, 112	0.12
Sex * FI	0.99	4, 112	0.42	1.01	4, 112	0.41
Condition * Sex * FI	2.01	4, 112	0.10	0.86	4, 112	0.49
**Evaluation of foods**						
Condition	2.62	4, 105	0.04^∗^	2.61	4, 105	0.04^∗^
FI	2.56	4, 105	0.04^∗^	2.49	4, 105	0.048^∗^
Sex	0.54	4, 105	0.71	0.52	4, 105	0.72
Condition * FI	1.25	4, 105	0.29	0.50	4, 105	0.73
Condition * Sex	0.97	4, 105	0.43	1.04	4, 105	0.39
Sex * FI	1.26	4, 105	0.29	1.81	4, 105	0.13
Condition * Sex * FI	2.09	4, 105	0.09	1.34	4, 105	0.26

The interaction between SSES condition and FI was not restricted to any one of the foods. In separate analyses of each outcome variable, the interaction term was significant or near-significant in all four cases (chocolate: *B* = 0.93, 95% CI −0.01–1.87, *t* = 1.95, *p* = 0.05; crackers: *B* = 1.22, 95% CI 0.35–2.09, *t* = 2.78, *p* = 0.006; crisps: *B* = 1.43, 95% CI 0.57–2.28, *t* = 3.31, *p* = 0.001; popcorn: *B* = 1.25, 95% CI 0.29–2.21, *t* = 2.58, *p* = 0.01). Using continuous AFI score instead of USDA as the FI measure, the significant interaction of FI and SSES condition was not found (Table 2). The main effect of condition was however significant.

Contrary to prediction, there were no interactions between FI and sex. Nor was there a significant main effect of sex on consumption.

### Evaluation of Foods

**FIGURE 2:**
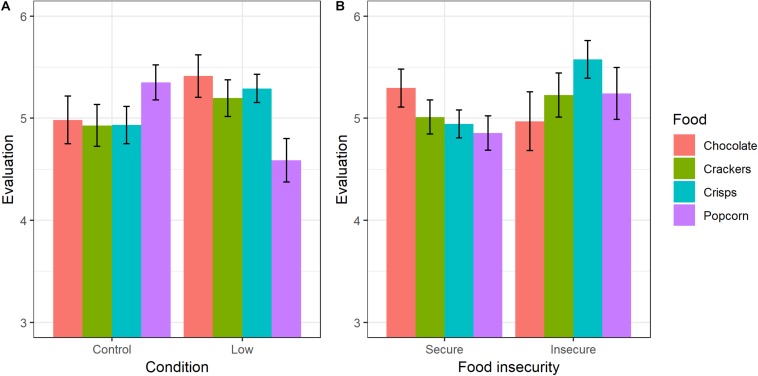
Mean evaluations of the foods: **(A)** by SSES condition and **(B)** by USDA food insecurity status. Error bars represent ±1 standard error.

For evaluation of foods, results were similar whether USDA or AFI was used as the FI measure: there were significant main effects of both condition and FI on the set of ratings, and no interaction (Table 2). The pattern across the individual foods was complex (Figure 2). The overall condition effect was driven by low SSES producing individually non-significant increases in the ratings of chocolate (*B* = 0.44, 95% CI −0.18–1.06, *t* = 1.41, *p* = 0.16), crackers (*B* = 0.26, 95% CI −0.28–0.80, *t* = 0.94, *p* = 0.38), and crisps (*B* = 0.33, 95% CI −0.11–0.77, *t* = 1.48, *p* = 0.14), but a significant decrease in the rating of popcorn (*B* = −0.78, 95% CI −1.32–0.24, *t* = −2.83, *p* = 0.005). The FI effect (results shown for USDA) was driven by food-insecure participants giving significantly higher ratings to crisps (*B* = 0.62, 95% CI 0.11–1.11, *t* = 2.44, *p* = 0.01), non-significantly higher ratings to crackers (*B* = 0.19, 95% CI −0.42–0.81, *t* = 0.63, *p* = 0.53), and popcorn (*B* = 0.42, 95% CI −0.19–1.03, *t* = 1.37, *p* = 0.17), but non-significantly lower ratings to chocolate (*B* = −0.34, 95% CI −1.03–0.35, *t* = −0.97, *p* = 0.33).

For the food-secure participants, we also tested, for each food, whether the increase in calorie consumption caused by the manipulation was mediated by changes to the ratings of the foods. In no case was the mediation effect significant (chocolate: *z* = −0.08, *p* = 0.45; crackers: *z* = 0.80, *p* = 0.43; crisps: *z* = 0.08, *p* = 0.19; popcorn: *z* = −1.83, *p* = 0.07). Likewise, for the control condition participants, we tested whether the effect of USDA FI on consumption was mediated by ratings of the foods. Again, there was no evidence of mediation (chocolate: *z* = −0.76, *p* = 0.30; crackers: *z* = 1.03, *p* = 0.30; crisps: *z* = 1.50, *p* = 0.13; popcorn: *z* = 1.59, *p* = 0.11).

### Positive and Negative Affect

Full analyses of the affect variables is provided in the Supplementary Material. In summary, being food insecure at baseline predicted higher negative affect and lower positive affect, but the manipulation was not significantly associated with either affect variable, either as a main effect or in interaction with FI. Affect variables did not predict food consumption or evaluation.

## Discussion

We measured consumption of freely provided snack foods, and the evaluation of those foods, in a sample of participants where baseline FI was known, and SSES was experimentally manipulated using a recently published method. Our first goal was to replicate the finding that experimental SSES manipulation affects consumption. We confirmed that it did. For the food-secure participants (who constituted the majority of the sample), the effect was as described in previous studies (Cardel et al., 2015; Bratanova et al., 2016; Cheon and Hong, 2016; Sim et al., 2018a): consumption was higher across the range of foods in the low status than the control condition. Our second goal was to replicate the finding that food-insecure participants consume more when given an opportunity to do so freely (Stinson et al., 2018; Nettle et al., 2019). Here, the replication was partial. In the control condition, food-insecure participants did indeed consume significantly more than food-secure participants. However, our prediction was that this would be true for the female but not male participants, and we found no interaction by sex. This could be a power issue given the unbalanced sex composition of the sample. Moreover, the effects involving FI were only significant using the USDA measure, not AFI. This does not appear to be a replicable pattern reflecting differences in what the two measures capture: in our previous comparable study (Nettle et al., 2019), the significant FI effects were found using AFI rather than USDA [though see Stinson et al. (2018) for significant FI effects using the USDA measure]. However, even correcting all *p*-values involving FI for the existence of two measures, the effects involving USDA FI would be deemed significant by conventional criteria.

Our most striking finding, however, was that FI and experimentally induced low SSES interacted strongly. It was not just that evoking SSES produced an increase in consumption only among food-secure participants, as might be expected if the SSES manipulation works by making the food-secure feel food-insecure. Rather, in food-insecure participants, consumption across the range of foods was significantly *reduced* in the low SSES condition compared to control. We did not predict the reversal *a priori*; there is the multiple-FI-measure issue mentioned above; and the number of USDA food-insecure participants was small (33). We therefore consider the finding exploratory and as requiring further investigation. As well as determining whether the interaction is robust, such investigation would need to explore why it occurs. It is possible that food-insecure participants are more explicitly concerned about their eating and weight, so that when faced with a negative status manipulation, they respond by over-riding an automatic tendency to increase eating, producing a deliberate over-compensation in the opposite direction. However, this remains a speculation on our part, since we had no measure of eating restraint or self-consciousness about eating and weight. Moreover, in this sample, those higher in FI did not have higher BMIs. We did measure negative and positive affect, but the only relationships of these measures to other study variables were that baseline food-insecure participants had higher levels of negative affect.

Food insecurity and low SSES were associated with differences in the evaluation of foods. However, this is only partially consistent with the hypothesis that the psychological mechanisms underlying FI and SSES effects on consumption involve changes in the hedonic value of food. This is because the changes in evaluation did not, for any of the foods, significantly mediate the FI or SSES effect on consumption, even though more positive evaluations of a food did weakly predict higher consumption of that food. Moreover, the effects of SSES and FI on evaluations were hard to interpret. Although the average rating across all four foods was higher in the low SSES condition than the control condition, and higher among food-insecure than food-secure participants, there was heterogeneity across the foods, with one food showing the reversed pattern in each case.

Although this study concurs with previous experimental results suggesting that experimentally manipulating SSES can alter patterns of eating and food motivation (Cardel et al., 2015; Bratanova et al., 2016; Cheon and Hong, 2016; Sim et al., 2018a), it has a number of limitations, beyond those of relatively small sample size and imbalance of the sample by sex and FI already noted. First, although the manipulation protocol we employed has now featured in a number of studies (Cheon and Hong, 2016; Sim et al., 2018b), no manipulation check is reported in those studies, and nor did we use one here. Thus, it remains to be established that what the manipulation does is specifically to change SSES; a priority for the field should be establishing that it does so. It is plausible that it would, and in this study the manipulation had no effect on general negative or positive affect. However, the magnitude, specificity, and time-course of its effects remain to be clarified. Moreover, neither this nor previous studies using the manipulation investigated the actual SES of the participants. The manipulation might have different effects on people of different SES, whose eating behavior might also be different. As our sample were mostly students, the actual SES range was likely to have been limited. Finally, even if SSES effects on short-term food consumption were shown to be robust, it would be naïve to assume that the causal link to obesity is thereby established. Short-term response to a standardized eating opportunity may be poorly related to habitual calorie consumption, and besides, calorie consumption is not related to weight gain in a simple way (Lucan and DiNicolantonio, 2015).

Nonetheless, experimental study of SSES effects on food motivation is important for theoretical understanding as well as, potentially, for interventions. While SES gradients in obesity have been documented for a long time, their causes are difficult to disentangle using epidemiological data (Jeffery and French, 1996). If changing how people perceive their status in society, without any change in their objective circumstances, can alter their eating, then we might come to different conclusions about what kinds of factors will and will not attenuate social gradients in diet and obesity. If the interaction between SSES and FI documented here proves robust, the same psychological intervention may have very different effects on different population sub-groups.

## Data Availability

The raw data from this study, along with R code used for analysis, are freely available via the Zenodo repository at: https://doi.org/10.5281/zenodo.2580973.

## Author Contributions

All authors conceived and designed the study collaboratively. SG and MR conducted the experimental sessions and collated the data. SG, MR, and DN performed the data analysis. SG and MR wrote the first draft of the manuscript. DN and MB revised the manuscript for content.

## Conflict of Interest Statement

The authors declare that the research was conducted in the absence of any commercial or financial relationships that could be construed as a potential conflict of interest.

## References

[B1] Anselme P. Güntürkün O. (2018). How foraging works: uncertainty magnifies food-seeking motivation. *Behav. Brain Sci.* 8 1–106. 10.1017/S0140525X18000948 29514723

[B2] Bickel G. Nord M. Price C. Hamilton W. Cook J. (2000). *Measuring food security in the United States: Guide to measuring household food security.* Washington, DC: USDA.

[B3] Bratanova B. Loughnan S. Klein O. Claassen A. Wood R. (2016). Poverty, inequality, and increased consumption of high calorie food: experimental evidence for a causal link. *Appetite* 100 162–171. 10.1016/j.appet.2016.01.028 26809142

[B4] Cardel M. I. Johnson S. L. Beck J. Dhurandhar E. Keita A. D. Tomczik A. C. (2015). The effects of experimentally manipulated social status on acute eating behavior: a randomized, crossover pilot study. *Physiol. Behav.* 162 93–101. 10.1016/j.physbeh.2016.04.024 27094920 PMC4899290

[B5] Cheon B. K. Hong Y.-Y. (2016). Mere experience of low subjective socioeconomic status stimulates appetite and food intake. *Proc. Natl. Acad. Sci. U.S.A.* 114 201607330. 10.1073/pnas.1607330114 27994148 PMC5224403

[B6] Goodman E. Adler N. E. Daniels S. R. Morrison J. A. Slap G. B. Dolan L. M. (2003). Impact of objective and subjective social status on obesity in a biracial cohort of adolescents. *Obes. Res.* 11 1018–1026. 10.1038/oby.2003.140 12917508

[B7] Goodman E. Adler N. E. Kawachi I. Frazier L. Huang B. Colditz G. (2001). Adolescents’ perceptions of social status: development and evolution of a new indicator. *Pediatrics* 108 1–8. 10.1542/peds.108.2.e31 11483841

[B8] Griskevicius V. Ackerman J. M. Cantú S. M. Delton A. W. Robertson T. E. Simpson J. A. (2013). When the economy falters, do people spend or save? Responses to resource scarcity depend on childhood environments. *Psychol. Sci.* 24 197–205. 10.1177/0956797612451471 23302295

[B9] Hill S. E. Rodeheffer C. D. DelPriore D. J. Butterfield M. E. (2013). Ecological contingencies in women’s calorie regulation psychology: a life history approach. *J. Exp. Soc. Psychol.* 49 888–897. 10.1016/j.jesp.2013.03.016

[B10] Jeffery R. W. French S. A. (1996). Socioeconomic status and weight control practices among 20- to 45-year-old women. *Am. J. Public Health* 86 1005–1010. 10.2105/AJPH.86.7.1005 8669502 PMC1380443

[B11] Lucan S. C. DiNicolantonio J. J. (2015). How calorie-focused thinking about obesity and related diseases may mislead and harm public health. An Alternative. *Public Health Nutr.* 18 571–581. 10.1017/s1368980014002559 25416919 PMC10271128

[B12] McLaren L. (2007). Socioeconomic status and obesity. *Epidemiol. Rev.* 29 29–48. 10.1093/epirev/mxm001 17478442

[B13] Nettle D. Andrews C. Bateson M. (2017). Food insecurity as a driver of obesity in humans: the insurance hypothesis. *Behav. Brain Sci.* 40:e105. 10.1017/S0140525X16000947 27464638 PMC5266557

[B14] Nettle D. Joly M. Broadbent E. Smith C. Tittle E. Bateson M. (2019). Opportunistic food consumption in relation to childhood and adult food insecurity: an exploratory correlational study. *Appetite* 132 222–229. 10.1016/j.appet.2018.07.018 30082103

[B15] Newton S. Braithwaite D. Akinyemiju T. F. (2017). Socio-economic status over the life course and obesity: Systematic review and meta-analysis. *PLoS One* 12:1–15. 10.1371/journal.pone.0177151 28510579 PMC5433719

[B16] R Core Development Team. (2018). *R: A Language and Environment for Statistical Computing.* Vienna: R Core Development Team.

[B17] Sim A. Y. Lim E. X. Forde C. G. Cheon B. K. (2018a). Personal relative deprivation increases self-selected portion sizes and food intake. *Appetite* 121 268–274. 10.1016/j.appet.2017.11.100 29170121

[B18] Sim A. Y. Lim E. X. Leow M. K. Cheon B. K. (2018b). Low subjective socioeconomic status stimulates orexigenic hormone ghrelin A randomised trial. *Psychoneuroendocrinology* 89 103–112. 10.1016/j.psyneuen.2018.01.006 29358119

[B19] Sobal J. Stunkard A. J. (1989). Socioeconomic status and obesity: a review of the literature. *Psychol. Bull.* 105 260–275. 10.1037/0033-2909.105.2.260 2648443

[B20] Stinson E. J. Votruba S. B. Venti C. Perez M. Krakoff J. Gluck M. E. (2018). Food insecurity is associated with maladaptive eating behaviors and objectively measured overeating. *Obesity* 26 1841–1848. 10.1002/oby.22305 30426695 PMC6249092

[B21] Townsend M. S. Peerson J. Love B. Achterberg C. Murphy S. P. (2001). Food insecurity is positively related to overweight in women. *J. Nutr.* 131 1738–1745. 10.1093/jn/131.6.1738 11385061

[B22] Wang X. T. Dvorak R. D. (2010). Sweet future: Fluctuating blood glucose levels affect future discounting. *Psychol. Sci.* 21 183–188. 10.1177/0956797609358096 20424042

[B23] Watson D. Clark L. A. Tellegen A. (1988). Development and validation of brief measures of positive and negative affect: the PANAS scales. *J. Pers. Soc. Psychol.* 47 1063–1070. 10.1037/0022-3514.54.6.1063 3397865

