# Supplementary material for: Food Insecurity Moderates the Acute Effect of Subjective Socioeconomic Status on Food Consumption
Source: Front Psychol. 2019 Aug 14;10:1886. doi: 10.3389/fpsyg.2019.01886 (PMC6702391; doi:10.3389/fpsyg.2019.01886)
Supplement: Supplementary file 1 [file Table_1.DOCX]

**Supplementary material for Godsell, Randle et al. ‘Food insecurity moderates the acute effect of subjective socioeconomic status on food consumption’**

**Analysis of positive and negative affect**

*Predictors of affect*

We first sought to establish whether the SSES manipulation and baseline FI predicted affective state. We fitted MANOVAs with the two PANAS affect variables as the outcome and FI, experimental condition and sex as the predictors (table S1). Negative affect was log transformed for analysis. Results were similar using USDA or AFI as the FI measure. There was a significant main effect of FI. Relative to food-secure participants, food-insecure participants had higher negative affect scores (USDA food secure: mean 14.67, sd 6.10; USDA food insecure: mean 19.15, sd 8.71) and lower positive affect scores (USDA food secure: mean 43.42, sd 10.39; USDA food insecure: mean 40.52, sd 8.79). In univariate analyses, the effect of FI was significant for negative affect (using USDA: t = 3.36, p = 0.001) not for positive affect (t = -1.43, p = 0.16). SSES condition was not significantly associated with the affect variables.

Table S1. Results of MANOVAs predicting affect variables.

|  | Using USDA FI status | | | Using AFI score | | |
| --- | --- | --- | --- | --- | --- | --- |
| Predictor | F | df | p-value | F | df | p-value |
| Condition | 1.03 | 2, 114 | 0.36 | 1.02 | 2, 114 | 0.36 |
| FI | 6.12 | 2, 114 | 0.003* | 4.59 | 2, 114 | 0.01* |
| Sex | 1.54 | 2, 114 | 0.22 | 1.63 | 2, 114 | 0.20 |
| Condition * FI | 0.93 | 2, 114 | 0.40 | 1.28 | 2, 114 | 0.28 |
| Condition * Sex | 1.13 | 2, 114 | 0.33 | 1.06 | 2, 114 | 0.35 |
| Sex * FI | 0.02 | 2, 114 | 0.99 | 0.58 | 2, 114 | 0.56 |
| Condition * Sex * FI | 0.78 | 2, 114 | 0.46 | 0.85 | 2, 114 | 0.43 |

*Affect and consumption*

We fitted a MANOVA with consumption of the four foods as the outcome, and positive and negative affect as the predictors (table S2). Neither affect variable significantly predicted consumption. We fitted a similar model with evaluation of the four foods as the outcome (table S2). Again, affect did not predict evaluation.

Table S2. Results of MANOVAs predicting consumption and evaluation of foods from affect variables.

|  | Consumption as the outcome variables | | | Evaluation as the outcome variables | | |
| --- | --- | --- | --- | --- | --- | --- |
| Predictor | F | df | p-value | F | df | p-value |
| Positive affect | 1.20 | 4, 117 | 0.32 | 1.38 | 4,110 | 0.24 |
| Negative affect | 1.26 | 4, 117 | 0.29 | 1.21 | 4,110 | 0.31 |
